# Gradient Legal Personhood for AI Systems—Painting Continental Legal Shapes Made to Fit Analytical Molds

**DOI:** 10.3389/frobt.2021.788179

**Published:** 2022-01-11

**Authors:** Diana Mădălina Mocanu

**Affiliations:** Centre for Philosophy of Law (CPDR), Institute for Interdisciplinary Research in Legal Sciences (JUR-I), Université Catholique de Louvain, Louvain-la-Neuve, Belgium

**Keywords:** AI systems, bundle theory of legal personhood, partial legal capacity, *teilrechtsfähigkeit*, gradient theory of legal personhood

## Abstract

What I propose in the present article are some theoretical adjustments for a more coherent answer to the legal “status question” of artificial intelligence (AI) systems. I arrive at those by using the new “bundle theory” of legal personhood, together with its accompanying conceptual and methodological apparatus as a lens through which to look at a recent such answer inspired from German civil law and named *Teilrechtsfähigkeit* or partial legal capacity. I argue that partial legal capacity is a possible solution to the status question only if we understand legal personhood according to this new theory. Conversely, I argue that if indeed *Teilrechtsfähigkeit* lends itself to being applied to AI systems, then such flexibility further confirms the bundle theory paradigm shift. I then go on to further analyze and exploit the particularities of *Teilrechtsfähigkeit* to inform a reflection on the appropriate conceptual shape of legal personhood and suggest a slightly different answer from the bundle theory framework in what I term a “gradient theory” of legal personhood.

## Prolegomena

“One cannot be too careful with words; they change their minds just as people do,” Nobel Prize–winning writer Jose Saramago once warned. “Words are events; they do things, change things,” Ursula le Guin, another distinguished writer and one of his devoted admirers, added. Nowhere else is this truer perhaps than in law’s empire^1^, where words such as “person” and “thing” are code for a “legal regime.” That is, they have the power to trigger a host of consequences once applied. In order to apply them, jurists qualify and categorize reality, thus establishing links between what is and what ought to be. The trouble with this attempt at fighting entropy by conceptually ordering reality is that the latter sometimes simply refuses to play by the rules that we set. This means that new entities in the world do not always fit our existing legal molds, and so we are faced with a conundrum: do we create new molds, or do we tweak and twitch (our understanding of) our entities to fit the old ones?

These very old such molds are currently being stretched to fit one very new type of entity, namely, AI systems^2^. Within the technical, as within the legal realm, word choice is crucial. Previously dubbed “autonomous artificial agents” or AAAs for short (Chopra and White, 2011) or “mathematically formalized information flows” (Teubner, 2018) and based on “artificial intelligence” (AI), to use a “suitcase word”^3^, AI systems exploit myriad increasingly complex and oftentimes opaque methods found at the intersection of computer science, statistics, and other fields to enable solutions that are adaptive in order to perform specific tasks with a degree of autonomy.

What all AI systems have in common is a set of features that make them straddle the border between “thing” and “person.” These are somewhat disputed, but most sources cite autonomy, usually related to a measure of agency, then adaptivity and self-learning. Embodiment is sometimes added to the list with claims that “genuine intelligence can emerge only in embodied, situated, cognitive agents” (Menary, 2007; Clark, 2017). This is lucky for robots but excludes a whole range of software-based entities. Adaptivity and self-learning abilities are sometimes reunited under “intelligence” (Kiršienė et al., 2011), another suitcase word with little to offer us in the way of a definition and perhaps even less so ever since it was coupled with “artificial” at Dartmouth in the 1950s. As it turns out, perhaps the most lasting contribution of this first attempt was not scientific, but semantic^4^.

The phrase “artificial intelligence” brings up questions about whether intelligent behavior implies or requires the existence of a (human) mind or to what extent consciousness, if real, is replicable as computation. Legally, properties such as consciousness have traditionally^5^ served as “qualifying criteria” (Gunkel and Wales, 2021) for natural personhood as opposed to the “artificial personhood” (Dyschkant, 2015) of entities such as corporations, which is said to be born out of the practical consideration of furthering some human interest more effectively. The term “intelligent machine” has recently been proposed as a metaphor for understanding both corporations and AI systems (Laukyte, 2021). Although somewhat unhelpful in cutting the Gordian knot due to the fact that we lack a definition for both intelligence or consciousness and that we have “epistemological limitations” (Gunkel and Wales, 2021) as to their detection in others, words and phrases such as these have been used by the law nonetheless, their vagueness making them the most debated points of contention in fringe cases on issues such as corporate rights, abortion, or euthanasia.

One of the few certainties we have is that AI systems are already widely used and will most likely infiltrate more and more aspects of everyday life, causing not just the way people think to be affected, but also the way they act and the manner in which they behave in their private and professional lives. This makes them legally significant because case-law that “anticipates the legal principles that may come to govern displacement of human activity by intelligent artifacts” (Wein, 1992) is bound to follow.

Because consensus is in general lacking as to whether legal innovation is in order, however, legal scholarship identified three criteria to determine when a new law is needed (Hondius, 1980). First, the legal problem has to fall either outside the scope of any existing branch of law or simultaneously under several branches, none of which resolves all aspects of the problem. Second, it has to affect broad sections of society and be likely to persist for a long time. Third, the new law has to result in basic principles sanctioned by the constitutional and legal system of the country concerned. Although this last one might prove problematic for legal innovations, because they mean changes to the very constitutional and legal systems by which they are supposed to be sanctioned, all three conditions are arguably met by AI systems’ legal “status question” (Papakonstantinou and de Hert, 2020).

## The Status Question

The status question (Schirmer, 2020) asks what exactly AI systems are, legally speaking. It makes us ponder whether we are just looking at sophisticated objects or things, whether we would rather treat them as legal persons, somewhat similar to humans or corporations, or indeed whether we should create a new legal category suited to their specificities. This way of phrasing the question mixes what exists in a material sense with what ought, from a moral point of view, to be and what we conventionally decide is or will legally be the case. Aside from the fact that we must exercise great care in juggling registers, because it is logically unsound and morally hazardous to derive an “ought” from an “is” (Norton and Norton, 2007) and slip from factual to axiological statements, this also reenacts to an extent the positivism versus natural law debate in the philosophy of law. Without becoming embroiled in the larger moral debate about AI systems as moral agents or patients, this contribution is strictly limited to law as artifactual, whether in order to refine expressions of some perfect idea of law (as natural law proponents hold) or simply to express renewed conventions (as positivists do).

The stark opposition between the two did not always exist as such though, and there might be a way around it too. Legal history shows us that legal fluidity coexists until it is gradually replaced by positive law, which is not immutable, but has to be adjusted to changes in society^6^ to not become obsolete or, worse yet, unjust, which makes the cycle repeat itself. An early but evocative example of this process would be the usage of *jus* by later Romans, whereas Cicero said *lex.* This is significant because the ambiguity of *jus* “lending itself to identification of what ought to be and what is, gave a scientific foundation for the belief of the jurisconsults that when and where they were not bound by positive law they had but to expound the reason and justice of the thing in order to lay down the law” (Pound, 1922). That is, natural law is an approach best suited for times of change, when jurists need to use their judgement to make analogies and, when that does not suffice, to create law to apply to new social realities. It is unsurprising in this light that a case for a natural law conception of AI legal personhood was made and assessed in the context of contemporary legislative proposals, concluding, however, that the time for creating such a concept is not ripe yet (Jowitt, 2021).

Creative periods of fluidity in legal history generally follow stable ones. As things stand today, it is difficult to find a more constant and undisputed legal assumption than the one underlying the conceptual framework of juridical humanism, widely accepted in Western legal systems and which rests on the dualistic division of legal reality into persons and things. I argue that this model of the world is an oversimplified one and that there is general skepticism to more inclusive change. This is beginning to change, however, since juridical humanism has been criticized as incoherent (Pottage and Mundy, 2004; Pietrzykowski, 2017), requiring a reconceptualization and reorganization of the relationships between these and new categories.

This incoherence stems from historical exclusions from the category of legal person of women and slaves and the inclusion of fringe cases, such as newborn children, differently abled adults, or animals (which is incongruous with the traditional definition of legal personhood) as well as (putative) attributions of personhood to rivers, idols, ships. For example, numerous European legal systems now explicitly exclude animals from the category of things, but there is no language as to a new category they may be part of although several suggestions exist, including “nonpersonal subjects of law” (Pietrzykowski, 2017) or “nonhuman (natural) persons” (Regad and Riot, 2018; Regad and Riot, 2020). Instead, positive law likens their treatment to that of goods, prompting legitimate complaints from animal rights activists about the purely formal “change” in status that practically amounts to as little protection and participation in legal life as before.

Change in the legal status of animals, let alone AI systems, is “not simply unacceptable, but rather unthinkable for many jurists” (Kurki, 2019)^7^. This is because the divide between persons and nonpersons is a part of the “deep structure of law” (Tuori, 2002), and questioning that binomial relationship is no easy feat. It has ample practical value though, and I argue it can be best accomplished through the logical and orderly analysis of law, which is the prime mission of the philosophy of law, or at the very least it is in its analytical bent. It bears noting at this point that the continental-analytical juxtaposition in the title can be misleading given that “continental” is used to refer to continental legal systems, and more specifically civil law, and not continental philosophy, whereas “analytical” does refer to the homonymous philosophical tradition. More specifically, continental legal shapes refer to the legal concepts of person and thing such as they exist in civil law traditions on the European continent. Analytical molds refer to the coherence of such legal concepts according to analytical methods.

Historically, philosophy “has been used to break down the authority of outworn tradition, to bend authoritatively imposed rules that admitted of no change to new uses which changed profoundly their practical effect, to bring new elements into the law from without and make new bodies of law from these new materials, to organize and systematize existing legal materials and to fortify established rules and institutions when periods of growth were succeeded by periods of stability and of merely formal reconstruction” (Pound, 1922). A method for such innovation has also already been proposed in relation to AI^8^, namely, conceptual engineering (Chalmers, 2020; McPherson and Plunkett, 2020). It holds that clarifying the content of core concepts should be the first step of any debate to avoid arguing about different things, but also to recover conceptual possibilities by figuring out what our concepts actually stand for and, more importantly, what they ought to stand for. We may, however, need to first engineer the concept of conceptual engineering itself, which is procedurally far from clear. This is in order to avoid a recursive engineering loop—a somewhat fitting irony given the context of application to AI systems—of both concept and method.

It is at any rate becoming increasingly urgent for law to take a stand in answering the status question for the case of AI systems. To do otherwise is to allow the possibility of “potentially impeding further development and the practical usefulness of the whole technology” (Pietrzykowski, 2017). Giving voice to the law on these matters should, however, aim to maintain or indeed establish the coherence of its discourse throughout. Therefore, in the interest of walking a moderate path, we would be ill-advised to legally tip the balance of power characteristic of the politics of nature (Latour, 2004) so irrevocably in our favor in relation to AI systems as to assume absolute responsibility for them (Kruger, 2021) without due consideration to the general question of whether AI systems should be regarded as being in our service or rather if the circle of legal subjects should be enlarged instead so as to include nonhuman entities.

To this question the EU seems to offer in answer its human-centric approach to AI regulation^9^. That law is an anthropocentric construction is fairly undisputed. It should perhaps come as no surprise that we have been tipping the scales of justice in our favor all along, in light of this premise. Human beings have made law preoccupied first and foremost with themselves and their wishes as to ordering lived reality. In an overt admission of speciesism, it is claimed (Bryson et al., 2017) that the main purposes of any human legal system revolve around giving preference to human material interests as well as human legal and moral rights and obligations over the similar claims of any nonhuman entities.

## Current Answers to the Status Question

Whether AI systems could be accorded (some form of) legal personhood, thus entering law’s ontology as legal persons is “a matter of decision rather than discovery” (Chopra and White, 2011). The same is true for qualifying AI systems as things however, and we gather that much, at least at a declarative level, from the EU Parliament’s apparent change of tune from the creation of “electronic persons” in 2017^10^ to its 2020 Resolution^11^. There is currently “no need” to give a legal personality to emerging digital technologies we are told in the latter. The initial ambition for a paradigm shift manifested in 2017 (Sousa Antunes, 2020) is, thus, wholly missing from more recent documents and that despite the fact that “moving past an anthropocentric and monocausal model of civil liability” was seen as a potential “unifying event” in a report^12^, which the commission had shortly before it tasked its Expert Group on Liability and New Technologies.

Subsequently, such a solution was not, however, seen as practically useful, mainly because “civil liability is a property liability, requiring its bearer to have assets” in order to give “a real dimension” to it, which would, in turn, require “the resolution of several legislative problems related to their legal capacity and how they act when performing legal transactions.” Despite acknowledging the fact that giving AI systems legal personality would not require including all the rights that natural or even legal persons have and that, theoretically, their legal personality could consist solely of obligations, such a step was still considered too much of a leap. It might well be, as opposed to just tinkering with traditional (liability) solutions while engaging in wishful thinking as to the harm caused by these technologies being reducible to risks that can ultimately be attributed to existing, albeit unidentifiable, natural or legal persons.

In the likely event of such attribution of harm to (legal) persons being hampered by too complex production chains or breaks in the chain of causality, affected parties do not have sufficient and effective guarantees of redress. Instead, we are simply told that “new laws directed at individuals are a better response than creating a new category of legal person” (Abbott and Sarch, 2019). Indeed, the tendency to rely on existing interpretations of the law instead of innovating is apparent in the European Commission’s Proposal for an AI Act as well (Veale and Zuiderveen, 2021).

The fact remains that humans or, shall we say, the natural persons who are taking the status decision, are inevitably bound to bias the answer toward safeguarding some human interests, especially given that AI systems currently do not (fully) display features traditionally considered as salient for the attribution of interests of their own. Which human interests get to be safeguarded is a balancing act that, for example, in the text of the 2020 Resolution arguably gave way to AI systems’ operators’ and emerging AI industries’ interests by softening risk-based liability. It introduced a two-tiered system of liability (with strict liability for high-risk systems and subjective liability with a presumption of fault for ordinary-risk systems) where only strict liability used to apply before. Indeed the creation and use of strict liability, or liability without fault, is linked with technological progress. What is more, the same text repeatedly warns against the overall increased risks involved in the operation of AI systems, which involve loss in control on the part of human operators. It seems counterintuitive in this light that “an increase in the risk factors indicated weakens the liability of the actor” (Sousa Antunes, 2020). Moreover, producers’ liability may not be amenable to similar differentiation into a two-tiered system, and while the fact that the two (i.e. operators’ and producers’ liability) need to articulate well for a liability regime to function well is undisputed, the question of how this articulation could function at all given this novel asymmetry remains unanswered as yet.

These mark only the beginning of a long string of pieces of legislation likely to tackle AI systems, however, and it would be premature to say that there has been a departure from the 2017 paradigm shift to the point of no return, especially given the phrasing “in the long run” utilized then to refer to the time frame of granting some form of legal personhood to “at least the most sophisticated robots” in order for them to “make good any damage they may cause”^13^. It has been argued (Papakonstantinou and de Hert, 2020) that liability is enhanced, not reduced, through granting legal personality to AI, the first and most important advantage of that being flexibility for every branch of law to assess the legal issues posed by AI systems within its own boundaries and under its own rules and principles, leading to tailor-made solutions as opposed to a “supervisory authority” with an opaque legal mandate to “monitor” any and all AI systems. Another advantage would be the proximity of one-to-one legal relationships with AI systems instead of the multitude of stakeholders involved in creating, operating, or putting them on the market, which, given modern production chains, are likely scattered all over the globe and prohibitively complex (Crawford, 2021).

The current piecemeal approach to regulating AI in the EU by identifying the sectors most likely to be affected by AI, highlighting potential problems and making concrete punctual suggestions for legislative intervention in order to address them “is in effect an amendment through *ad hoc* patches” (Papakonstantinou and de Hert, 2020) of the legal framework currently in effect using existing legal tools. It might amount to a change in legal status nonetheless, given enough tinkering, but a formal recognition of that would still need to come either *via* case law decided by the Court of Justice of the European Union or positive law.

## The Case for Legal Creativity

The legal nature of AI systems is a preoccupation arising within “legal reality” (Hermitte, 1999), an environment that purports to organize and, thus, help make sense of lived reality, in which technology evolves in ways that challenge our legal models. We are, thus, faced with having to breach a gap that, in the long run, will presumably only deepen. Pragmatically, their functionality and social role as well as our relationships with AI systems will probably be the decisive arguments to sway the answer to the status question. The economic context was even said to lead to changes in the status of AI systems before that of nonhuman animals (Michalczak, 2017).

Arguing that AI systems may have potential legal subjectivity based on an analogy to animals, however, or even juristic persons for that matter, superficially suggests “the existence of a single hierarchy or sequence of entities, organized according to their degree of similarity to human beings” (Wojtczak, 2021). The place of an entity in this hierarchy would determine the scope of subjectivity attributed to it, a subjectivity that would be “derivative” in nature and not different from that of animals and companies.

Subjecthood could instead become a sort of master mold. Diversifying status thus, we would create the all-encompassing meta-category of subjects, including persons and other “nonpersonal subjects” (including human nonpersonal subjects and extra-human nonpersonal subjects). Nonhuman animals were already used as an example of beings whose legal status could be changed from things to “nonpersonal subjects”—not quite legal persons, but not things either (Pietrzykowski, 2018). Such subjects would, according to this opinion, differ from traditional persons in that they would be the holders of limited rights, or—in the case of animals—the single subjective right to be taken into account and have their interests duly considered and balanced whenever legal decisions affect them.

To further complicate matters, in many European languages, the term or phrase “legal subject” or “subject of law/right(s)” (*Rechtssubjekt*; *sujet de droit*) already is an umbrella term, referring to both natural and artificial persons, i.e., individual human beings and corporations or other such associations, respectively. This usage of *Rechtssubjekt* was introduced by Savigny. Civil law jurisdictions use the phrase “legal subject” or “subject of law” when addressing legal persons, whereas such phrases may seem odd to common lawyers, who use legal person to refer to artificial persons (Kurki and Pietrzykowski, 2017).

**FIGURE 1:**
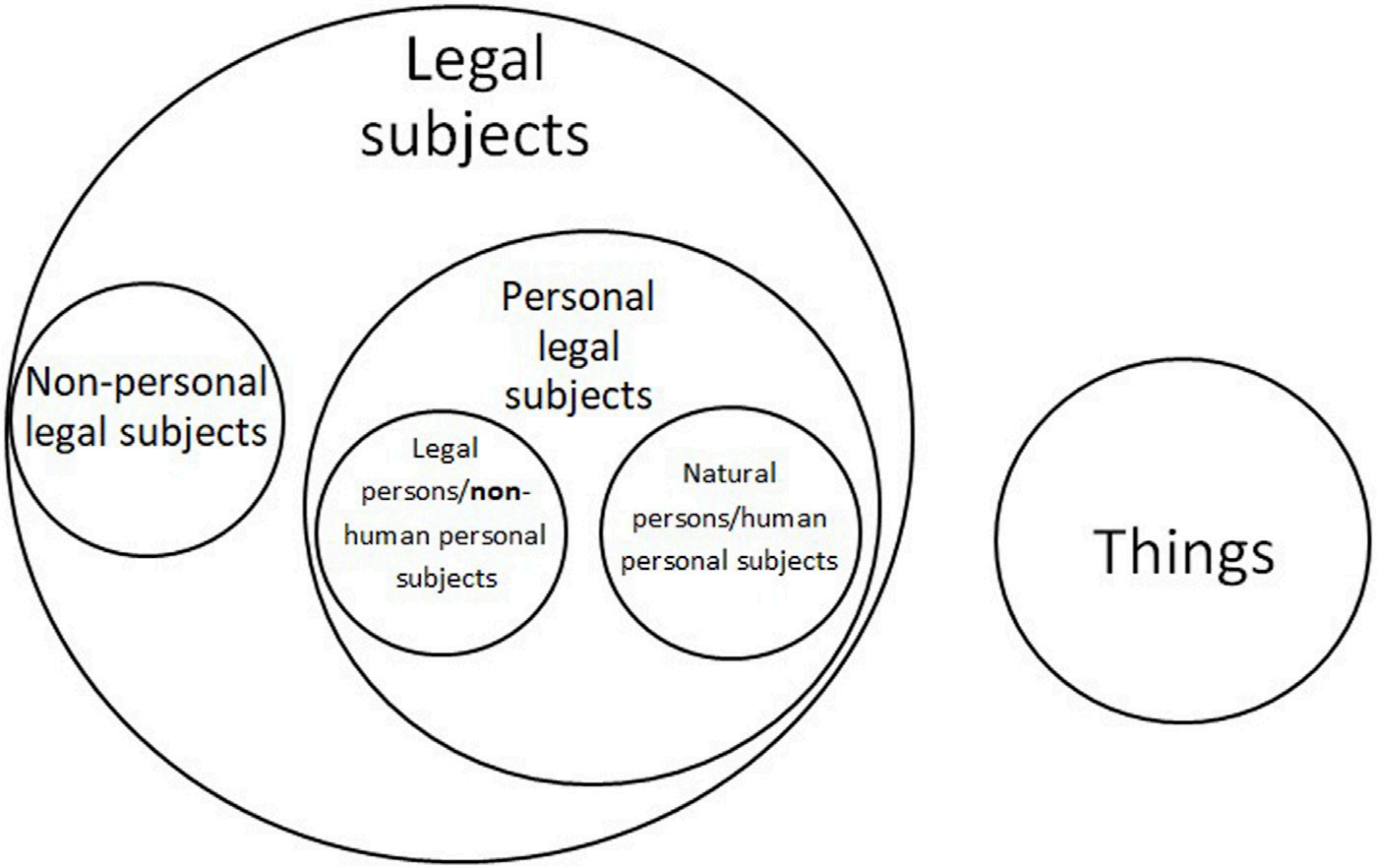
Diversifying legal status.

This begs the question of how exactly a Venn diagram might show the relationships between these concepts. We could for instance, looking at the diagram in Figure 1, imagine the circle of “things” intersecting that of “non-personal legal subjects” and therefore the larger “legal subjects” one. It also allows us to question in which of the categories illustrated below AI systems might end up included, or indeed whether an entirely new category might be defined specially to include them.

What thus becomes paramount is delimiting the extension of the concept of legal personhood, the attribution of which has in legal theory been thought of as either requiring certain preexisting conditions, or not requiring any at all. If it does not, then it is in this case merely a fiction^14^, an instrument of law or a label that we apply to trigger certain consequences, which can be either predetermined in bulk or established on a case-by-case basis. If it does, however, require certain preexisting conditions, then the debate moves to determining what these may be. Needless to say, there is no consensus.^15^

Either way, translating what is into what is in legal terms so that we can compare it against what we think ought to be and act on that basis to establish or restore some sense of balance is what jurists of all times have trained to do. It is also what they obsess about to such a degree that the is–ought distinction becomes blurred at times. To repeat a rather amusing metaphor for this confusion, “when a jurist gets bitten by a dog, they do not scream, but think about whether the conditions for liability for the animal’s action are met” (Rizoiu, 2020). What if, instead, the jurist suffers harm as a consequence of the behavior of Boston Dynamics’ dog-like robot, Spot? It was argued that facilitating liability mechanisms for holding AI systems directly liable for (at least some of) the effects of their decisions and actions is one of the most compelling arguments in favor of granting “sufficiently complex” AI systems “some form of personhood instead of regarding them as ordinary things (mere machines)” (Solum, 1992; Chopra and White, 2011). Whereas the possibility of applying “electronic personality” to them has been vastly criticized^16^, the critiques going in this direction generally rely on a concept of legal personhood that is—it has been successfully claimed, as we shall see—in need of a reappraisal (Kurki, 2019).

## Legal Personhood *Qua* Bundle

The bundle metaphor is used to depart from the “orthodoxy” (Kurki, 2019) of legal personhood as the capacity to hold rights and duties, explaining it instead as a cluster of interconnected “incidents.” As a “cluster property” or a property “whose extension is determined based on a weighted list of criteria, none of which alone is necessary or sufficient”^17^, legal personhood could have different configurations, mirroring different legal contexts. Legal personhood cannot be equated with the holding of rights because modern theories of rights, which are based on Hohfeld’s conceptual clarifications on the notion of “right”^18^, “either ascribe rights to entities that are not usually classified as legal persons, such as foetuses and nonhuman animals, or deny rights to entities that are ordinarily classified as legal persons, such as human children” (Kurki, 2019). There are glaring discrepancies between the list of holders of rights and obligations according to contemporary theories about the foundations of subjective rights and the list of persons according to the much older “orthodox view” on legal personhood, although they should be identical if the latter had an adequate definition.

Because “paradigmatic doctrinal judgements” and “extensional beliefs” about who or what constitutes a legal person would be nearly impossible to change and would, even if changed, offer little in the way of an explanation, what follows is that the definition of legal personhood should be adapted to accommodate modern theories of rights (Kurki, 2019). The “bundle theory” offers one such adaptation, using reflective equilibrium as a method^19^.

For the specific case of AI systems, the bundle theory is used to analyze three contexts that influence the outcome of the debate over their legal status, namely “the ultimate value context” (whether artificial intelligence has intrinsic moral value, not derived from its usefulness to other entities), “the liability context” (whether artificial intelligence can be held tortuously or criminally liable for its actions), and last but not least “the commercial context” (whether artificial intelligence can function as a commercial actor).

The first of these three, ultimate value, is connected to passive legal personhood, which “functions through claim-rights” (Kurki, 2019), allowing correlative duties to be owed to AI systems. Even if an AI is of ultimate value, we are left without an explanation as to why this is relevant for status ascription *per se* so much as with the impression of the question being moved to the territory of ethics. If an AI is not of ultimate value, however, and this seems to be the case according to the bundle theory, then it can only hold claim rights as the administrator of a human-defined project. The only avenue left for them to be legal persons in that case runs through their “capacity to be subjected to legal duties and/or to administer legal platforms through the exercise of competences” (Kurki, 2019)*.*

A “legal platform” refers to the legal positions held by a “legal person” and was introduced in the bundle theory to distinguish the two as well as to counterbalance the systematically ambiguous current doctrinal use of “legal person” to refer to both. That AI systems can hold claim rights as administrators of legal platforms with objectives set by human beings was concluded elsewhere too (Bayern, 2021), using similar examples to those utilized here, of an investment bank that hires trader bots to buy and sell stocks at a superhuman pace and a foundation run by an entity based on artificial intelligence.

In the commercial context, another distinction is superposed, between independent and dependent legal personhood based on the amount of supervision necessary in the exercise of competences by AI systems, which would place them on a tool–representative–legal person continuum.

Attaching legal platforms to entities that do not fulfill certain criteria would prove useless according to the bundle theory. The ability to hold claim rights makes for passive legal personhood and the ability to perform legal acts for active legal personhood. Passive legal personhood designates legal capacity and is likened to *Rechtsfähigkeit* or *capacité de jouissance* in German and French continental law, respectively, whereas active legal personhood corresponds to legal competence, *Geschäftsfähigkeit* or *capacité d’exercice*. These parallels to civil law traditions prove useful for analyzing a recently proposed solution to AI systems’ legal status question, namely, *Teilrechtsfähigkeit* or partial legal capacity (Schirmer, 2020).

## The Partial Legal Capacity Variation

This ontological category of legal subjects, halfway between person and object^20^ was inspired from German civil law and dubbed “a half-way status” or “a status of partial legal subjectivity based on certain legal capabilities.” Partial legal capacity would entail treating AI systems as legal subjects as far as this follows their alleged function of “sophisticated servants” (Schirmer, 2020).

Juridical humanism’s all-or-nothing version of legal personhood is ill-suited for explaining such flexibility, which, in turn, seems to confirm the bundle theory. Born out of a critique of the two-tier system of legal capacity as inconsistent with the reality of how legal systems treat minors or used to treat women and slaves, partial legal capacity is a later materialization of the conclusion that legal capacity comes in plurals and there are, accordingly, many legal statuses. Defined in the 1930s as a status applicable to a human or an association of humans having legal capacity only according to specific legal rules but otherwise not bearing duties and having rights, it is, thus, an expansion of our understanding of legal capacity.

Although bent out of form and used for the practical disenfranchisement of the Jewish population^21^, it survived *via* court judgments regarding the unborn or preliminary companies. In German law, the preliminary company (*Vorgesellschaft*) is considered a legal entity of its own kind (*Rechtsform sui generis*) subject only to the rules of the articles of association and the statute governing the company, insofar as those laws do not require registration. This also applies to certain company types such as the company constituted under civil law or the homeowner’s association.

In the case of the first two, *i.e.,* the unborn and preliminary companies, the use case covered by partial legal capacity seems to be concerning entities “in the making.” In this sense, it is a transitional state. The temporal and temporary dimension is more evident in some civil law jurisdictions than in others. For example, in Romanian civil law, the preliminary company enjoys “anticipatory legal capacity”^22^ or limited legal capacity to perform the necessary legal acts in anticipation of its own formation. Article 60 of the Belgian *Code des sociétès* on the other hand sets an “imperfect liability,” meaning that natural persons acting on behalf of the company (such as the founders) engage its personal liability in performing acts necessary for founding the company, such as renting an office. Once a legal person has been created, it takes on the contract itself, in a postconstitutive transfer of full liability. Here, the coherence of the institution of contracting is at stake. The use of the term “imperfect” denotes the same transitional state mentioned prior, which the law struggles to accommodate.

In the case of the latter examples, i.e., the company constituted under civil law or the homeowner’s association, it is a question of the specific assortment of rights and duties attributable to a human or an association of humans having legal capacity only according to specific legal rules. This might refer perhaps to the law’s presuppositions about the legal person in its various subdomains as it does about the diligence and reasonableness of the *bonus pater familias* wearing their administrator *persona* and which points, in turn, to an intrasystem asymmetry (Novelli et al., 2021) as to the meaning of personhood in different legal subfields. It might, on the other hand, refer to the limiting principle of specialization that circumscribes the legal capacity of juristic persons such as companies around its object as formulated in its statutes. The problem with this is that, in general, statute formulations are so vague and encompassing as to prevent any legal challenge based on an alleged *ultra vires* act performed in the company’s name.

In terms of partial legal capacity, accretions of rights need to be justified according to the function of the entity in question, and the only binding expression of that function is, in the case of companies, their statute. In the case of AI systems, as we shall see, functionality is largely inscribed in the artifact but should also be formalized to avoid misuse or abuse. A way of inventorying their functions and necessary capacities to accomplish such functions so that abuse is kept in check would be *via* registries. Preliminary companies, however, must not be subject to registration if they are to possess partial/anticipatory/imperfect legal capacity and be considered legal entities of their own kind, which is to say that humans decide what legal persons they inventory.

At any rate, partial legal capacity does not work by limiting capacity, but by allocating or adding legal capacities as they are justified, as opposed to legal personhood, which asks us to justify their subtraction. This is how partial legal capacity is supposed to, solve the slippery slope of having to justify denying worker and constitutional rights to AI systems, which is one of the “negative side effects of full legal personhood” being attributed to these entities (Schirmer, 2020). Seen through the lens of the bundle theory and the above examples, partial legal capacity could actually amount to personhood, albeit as a smaller bundle.

These examples do not show legal persons with full legal capacity, but they do show legal subjects nonetheless, though with the range of their subjectivity limited by their specific functions. This characterization joins the bundle theory’s assertion that there are several ways in which the law might treat entities in the world “more or less as persons” (Kurki, 2019)*.* It might do so for a particular purpose and not others, it adds, pointing to the general variety of the law’s purposes and the corollary flexibility required of legal personhood for it to better suit them. It leaves some doubts, however, as to the nature of the conceptual relationship between function, purpose, and competence with the latter taking center stage when the bundle theory is applied to the case of AI systems in the commercial context as we have seen.

Indeed, function and purpose seem to commingle in the rights theory and theory of personhood registers. A possibility would be to think of function as a binder between the more abstract “purpose” and the concreteness of “competence.” It could, thus, serve as an intermediary, negotiating the proper shape of personhood between what AI systems can and should do and what we can and should make them do. This functionalist approach is, therefore, not task oriented *per se* but shifts the focus from the technical capabilities that AI systems are designed to have to the things that they are made to do for humans. In other words, the problem is put in terms of a relational approach (Coeckelbergh, 2010; Gellers, 2021). Moreover, AI systems are communicative entities, and even in moving away from considering communication as the relevant criterion for the personification of nonhuman entities, we should still consider it as relevant to whether they should be treated more like persons (Darling, 2016; Darling, 2021), including legally, since it makes us perceive them as life-like.

Communication is certainly important from a legal perspective, not least because it is what makes possible voicing internal mental states, on the expression of which rests the foundation of legal responsibility attribution. Thus, ascribing legally binding intentions to AI systems as communicative entities has been explained *via* a systems theory generalization of the “intentional stance”^23^. Intentionality is fundamental to contracting and, combined with the pervasive objectivization tendencies in contract theory springing from technological advancement, amounts to the possibility for “software agents” to make legally effective declarations of intent (Teubner, 2018) as opposed to just being a prolongation of the creators’ intention. As we have already seen, however, this fails to account for the passive aspects of legal personhood as well.

## Legal Personhood *Qua* Gradient

As the bundle theory unfolds, it becomes increasingly clear how such an account of personhood as a cluster concept can be mobilized with ease in fringe cases, in which not all incidents of legal personhood are at stake. This also makes the conceptual borders of personhood rather more blurred, however, raising the issue of salient criteria and thresholds and inviting reflection on whether it might not be vulnerable to a sorites paradox critique^24^. In other words, it invites the question of what makes a bundle. Because “bundle” has unclear boundaries it seems that no single incident added or subtracted can make a difference between a bundle and a nonbundle, and therefore, the threshold to legal personhood seems rather arbitrary.

The bundle metaphor has connotations of artificially tying together a set of nondistinct or random items, whereas a gradient might be a metaphor more apt at capturing the quality of legal personhood as a cluster property with its extension determined based on a weighted list of neither necessary, nor sufficient criteria. This, in turn, suggests different items of the same kind (in this case rights) can be added or subtracted to end up placed differently on the gradient, much like in the case of the RGB or CMYK color models for instance.

As a gradient, legal personhood is not, therefore, only a matter of adding or subtracting from a bundle of legal incidents with a minimum threshold below which we can no longer call it a bundle, but it also takes into account the kinds of items added or subtracted so that an entity can be a legal person for some specific purposes only, as in the partial legal capacity example, in which function plays a central role. This is reminiscent of the origins of the concept of legal personhood in the mask worn by ancient Greek actors on stage and that came to represent the different roles played by a person in the many areas of life and law. Vendor, partner, accused, administrator, or reasonable person are all masks one wears, sometimes superimposed, but always molded to fit them and whatever the norms of the day demanded for their protection and participation to legal life.

A loose parallel becomes possible here with David Hume’s bundle theory of personal identity and the self, according to which “the peculiarly complex unity or identity of the self should be interpreted in terms of constantly changing causal relations, more like the identity of a complex play than a simple material object”^25^. What serves as inspiration here, however, is rather the gradient theory of personal identity recently attributed^26^ to Anne Finch Conway (Gordon-Roth, 2018), whose views suggest a spectrum of creatures distributed in a kind of personhood gradient in which some are more or less of a person than others. A certain threshold is envisageable, but necessary conditions for passing it are not. Only sufficient conditions might be, and research to uncover the subtleties of this view is on-going.

Regardless of whether we choose to think of personhood as a bundle or as gradient, the important premise remains that legal personhood is a complex attribute in legal theory, having been expressly characterized as “gradable” aside from it also being “discrete, discontinuous, multifaceted, and fluid” (Wojtczak, 2021). This because it can contain a variable number elements of different types—such as responsibilities, rights, competences, and so on—which can be added or taken away by a lawmaker in most cases with some notable exceptions, chiefly concerning the natural personhood of humans, who cannot be deprived of their human rights, and neither can they renounce certain subjective rights. This (conveniently) mirrors the existence of thresholds posited philosophically and is also a common point between the bundle and gradient approaches to legal personhood. Both reject that anything goes when it comes to legal personhood, the latter based on the worry that such a legal instrument, malleable in the extreme, would ultimately become meaningless and ineffective in its declared purposes of protection and participation in legal life.

## Conclusion

AI systems are in a rather singular position. We are making them show us reality in novel ways, and they are making us reconsider the way we order it in return. No matter how we formulate our answer to the legal status of AI systems question, it must acknowledge the fact that law is artifactual. Being much more in line with what we think of as such, molds are yet another helpful metaphor. They are not mere collections of things tied together by the proverbial *vinculum juris*, but tools for creating new things altogether, extensions of composing parts with their shape, size, and color situated on gradients.

Given that the skills involved in making such tools were acquired only of late by this hybrid between *homo faber* and *homo juridicus* that *homo sapiens sapiens* seems to be, they need honing. Engineering complex concepts, such as legal personhood can be looked at as a work in progress from this perspective. Applying them to such uncanny novel entities as AI systems requires the use of every other available tool in the analytical toolbox to fashion a smooth transition in the face of the overwhelming changes brought about by the advent of AI.

The underlying assumption being that AI systems’ legal status is a matter of utmost importance because it determines which law is applicable and enforceable as to their uses and the ensuing consequences of those uses, this article proceeds to deconstruct that assumption by first looking at why there is a status question concerning these entities in the first place. It then inventories the possible answers to that question according to the currently entrenched legal theoretical framework and makes the case for legal creativity when it comes to the options available as to status ascription to better fit the uncanny entities that AI systems are. It then looks at methods for so doing and details one particular recent approach to solving the problem by reconceptualizing legal personhood as a bundle, which is the state of the art in our theoretical understanding. Through this new lens, it goes on to analyze “partial legal capacity” recently proposed as a solution to AI systems’ legal status question. It concludes that accepting it means accepting the bundle theory of legal personhood or, at the very least, accepting that legal personhood is a cluster concept. Finally, it suggests, upon further analysis, that framing it in terms of gradient might be better suited to explain at least some use cases, AI systems included. It, therefore, sketches some incipient ideas on what could, with further research, perhaps develop into a gradient theory of legal personhood.

These theoretical adjustments are necessary and significant for a more coherent answer to the legal status question of AI systems. Such an answer, well-grounded in legal theory, has the potential to influence the future legal treatment of AI systems. It can also help judges decide the hard cases involving AI systems with which they will undoubtedly be faced, not to mention help lawyers argue such cases. Perhaps most importantly, however, if such a theory succeeds in painting a clearer picture of all the relevant facets of the legal issues at stake, it could contribute to better balancing the interests of all those involved.

## Data Availability

The original contributions presented in the study are included in the article/Supplementary Material, further inquiries can be directed to the corresponding author.

## Footnotes

1 To echo Ronald Dworkin in *Law’s Empire*, Belknap Press, 1986, whose fictitious judge Hercules would perhaps be ideally situated to undertake the work of legal interpretation and hermeneutics required to properly situate new technologies in the legal domain.

2 I use the term “AI systems” throughout this paper, borrowing it from the European Commission’s Proposal for a Regulation laying down harmonized rules on artificial intelligence (Artificial Intelligence Act), fully aware of the criticism directed at the too broad definition thereof. This choice is motivated by the fact that the author of the bundle theory of legal personhood expressly avoids reference to AI systems as “agents” or “actors” or even “actants” to underline the fact that legal personhood can be purely passive as with infants. More details on passive legal personhood can be found in section 5.

3 Marvin Minsky, American mathematician, computer scientist, and AI pioneer, in an interview on the subject of his book *The Emotion Machine*, Simon and Schuster, 2006, conducted by John Brockman for Edge.org and titled *Consciousness is a big suitcase: A Talk With Marvin Minsky*, available at: https://www.edge.org/conversation/marvin_minsky-consciousness-is-a-big-suitcase. Interestingly, in his previous book, *The Society of Mind*, Simon and Schuster Paperbacks, 1988, he used “agents” to refer to the units of his “society of mind,” the metaphor employed to explain how intelligence and a mind are accretions of different combinations of rather unintelligent and mindless composing units.

4 Roberts, J. (2016). Thinking Machines: The Search for Artificial Intelligence - Does History Explain Why Today’s Smart Machines Can Seem So Dumb? Distillations magazine, Summer issue. Available at: https://web.archive.org/web/20180819152455/https://www.sciencehistory.org/distillations/magazine/thinking-machines-the-search-for-artificial-intelligence.

5 Recently, however, support against the idea that the criteria of legal subjectivity are consciousness, sentience or reason has been amassing. See for example Gunther Teubner, *op.cit.,* who proposes communication as the salient criterion while citing numerous other criteria proposed; for an even more recent contribution see Sylwia Wojtczak, *op.cit.*, proposing participation or presence in social life as the salient criterion.

6 Pound offers a very early example of such coexistence, which comes to us in the form of an exhortation addressed by Demosthenes to an Athenian jury, saying that “men ought to obey the law for four reasons: because laws were prescribed by God, because they were a tradition taught by wise men who knew the good old customs, because they were deductions from an eternal and immutable moral code and because they were agreements of men with each other binding them because of a moral duty to keep their promises.” Modern legal eyes might just dismiss these four reasons as contradictory, but that would be ignoring the fact that they served a very practical purpose of establishing social control in a primitive society by whatever means necessary. The classical Greeks were just then trying to cement some basis of authority for law, which we today largely take for granted although we still question whether right is right by nature or by convention. That would also be ignoring another factor in the establishment of social control through law, namely, that it takes time.

7 David Gunkel has recently mapped where authors having written on the subject of robot rights fall on that debate, with unthinkable associated to names such as Noel Sharkey, Luciano Floridi, Alan Winfield, Sherry Turkle, Abeba Birhane, or Jelle van Dijk.

8 Köhler, S., and Himmelreich, J. (2021). Responsible AI through Conceptual Engineering, Talk Given for TUDelft's. AiTech Agora.

9 The term is part and parcel of the recent European Commission Proposal for an AI Act mentioned previously, published on April 21, 2021 and available at: https://ec.europa.eu/newsroom/dae/document.cfm?doc_id=75788.

10 European Parliament (2017) Resolution of 16 February with recommendations to the Commission on Civil Law Rules on Robotics [2015/2103(INL), sec. 59 f] states that “in the long run, it will be necessary to create a specific legal status (. . .), so that at least the most sophisticated autonomous robots could be established as having the status of electronic persons responsible for making good any damage they may cause, and possibly applying electronic personality to cases where robots make autonomous decisions or otherwise interact with third parties independently.”

11 European Parliament, Report with recommendations to the Commission on a civil liability regime for artificial intelligence (2020/2014(INL)), available at: https://www.europarl.europa.eu/doceo/document/A-9-2020-0178_EN.html.

12 Expert Group on Liability and New Technologies, New Technologies Formation, *Liability for Artificial Intelligence and other emerging digital technologies*, p. 19, available at: https://ec.europa.eu/transparency/regexpert/index.cfm?do=groupDetail.groupMeetingDoc&docid=36608.

13 European Parliament, Resolution of February 16, 2017, with recommendations to the Commission on Civil Law Rules on Robotics (2015/2103(INL)).

14 Variations of this view are called legalist, with the so-called “anything-goes approach to legal personhood” attributable to Natalie Stoljar by Ngaire Naffine in *Who are Law’s Persons? From Cheshire Cats to Responsible Subjects*, 66 Modern Law Review, 2003, pp. 346–351.

15 We owe the legalist realist distinction to Ngaire Naffine too. Reason has ample support as the salient feature, but, religionists tell us it is the soul, and naturalists deem it to be sentience. All of these are variations of the so-called realist view.

16 Open letter to the European Commission Artificial Intelligence and Robotics, signed by almost 300 professionals and experts in the relevant fields, available at: http://www.robotics-openletter.eu/.

17 See John R. Searle, *Proper Names*, (1958) 67 *Mind* 166. What *is* necessary and sufficient is a disjunction of certain proper subsets of the set of cluster properties.

18 Wesley Newcomb Hohfeld’s writings are few because of his too short life, but nonetheless important contributions to legal theory: *The Relations Between Equity and Law*, 1913, Michigan Law Review, 537; *Some Fundamental Legal Conceptions as Applied in Legal Reasoning*, Yale Law Journal vol. 23, no. 1/1913, pp. 16-59; *Fundamental Legal Conceptions as Applied in Judicial Reasoning*, Yale Law Journal vol. 26, no. 8/1917, pp. 710-770.

19 Known to us from John Rawls’ writings.

20 See Peter H. Kahn, Jr., et al., *The New Ontological Category Hypothesis in Human-Robot Interaction*, 2011 PROC. 6TH INT’L CONF. ON HUMAN-ROBOT INTERACTION 159; Ryan Calo, *Robotics and the Lessons of Cyber law*, 103 Calif. L. Rev. 513 (2015), https://digitalcommons.law.uw.edu/faculty-articles/23.

21 According to Schirmer, this view of legal capacity as plural and governed only by specific legal rules that do not give rise to rights and obligations served as justification for the gradual subtraction of rights from the Jewish population leading up to the second World War.

22 Article 205, (3), Romanian Civil Code states (*trad.n*.), “However, the legal persons dealt with in paragraph (1) (subject to registration) can, starting at the date of their constitutive act, acquire rights and take on obligation, but only insofar as they are necessary for the valid creation of said legal persons.”

23 “The intentional stance should be adopted when the behavior of a system is best explained and/or predicted if we attribute beliefs and desires to that system,” according to Daniel C. Dennett, *The Intentional Stance*, The MIT Press, Cambridge, Massachusetts, first published in 1987, freely available online courtesy of the Internet Archive at https://archive.org/details/intentionalstanc00dani.

24 Hyde, Dominic and Diana Raffman, “Sorites Paradox”, The Stanford Encyclopedia of Philosophy (Summer 2018 Edition), Edward N. Zalta (ed.), available at: https://plato.stanford.edu/archives/sum2018/entries/sorites-paradox/.

25 According to the entry on “the bundle theory of the self” of David Hume, *A Treatise of Human Nature*, i. iv. 6, from Ted Honderich (ed.), *The Oxford Companion to Philosophy*, second edition, Oxford University Press, 2005.

26 Alex Jensen, *Conway and Locke on Personhood*, detailing Heather Johnson’s research as part of a project to diversify the cannon under way at the University of Minnesota, available at https://cla.umn.edu/philosophy/news-events/story/conway-and-locke-personhood.

## References

[B1] Abbott R. Sarch A. F. (2019). Punishing Artificial Intelligence: Legal Fiction or Science Fiction. UC Davis Law Review. 10.2139/ssrn.3327485

[B2] Bayern S. (2021). Autonomous Organizations. Cambridge University Press.

[B3] Bryson J. J. Diamantis M. E. Grant T. D. (2017). Of, for, and by the People: the Legal Lacuna of Synthetic Persons. Artif. Intell. L. 25, 273–291. 10.1007/s10506-017-9214-9

[B4] Chalmers D. J. (2020). What Is Conceptual Engineering and what Should it Be? Inquiry.

[B5] Chopra S. White L. F. (2011). A Legal Theory for Autonomous Artificial Agents. Ann Arbor: University of Michigan Press.

[B6] Clark A. (2017). “Embodied, Situated, and Distributed Cognition,” in A Companion to Cognitive Science. Editors Bechtel, W. Graham G. (Wiley). 10.1002/9781405164535.ch39

[B7] Coeckelbergh M. (2010). Robot Rights? towards a Social-Relational Justification of Moral Consideration. Ethics Inf. Technol. 12, 209–221. 10.1007/s10676-010-9235-5

[B8] Crawford K. (2021). Atlas of AI. Yale University Press.

[B9] Darling K. (2021). The New Breed: What Our History with Animals Reveals about Our Future with Robots. New York: Henry Holt and Company.

[B10] Darling K. (2016). “Extending Legal Rights to Social Robots, SSRN Journal,” in We Robot Conference 2012. Editors Froomkin C. eds. K. Law R. Elgar E. (Cheltenham, UK; Northampton, MA, USA: University of Miami). 10.2139/ssrn.2044797

[B12] Dyschkant A. (2015). Legal Personhood: How We Are Getting it Wrong. University of Illinois Law Review, 2075–2110.

[B46] Gellers J. C. (2021). Rights for robots. Artificial Intelligence, Animal and Environmental Law. New York: Routledge

[B13] Gordon-Roth J. (2018). What Kind of Monist Is Anne Finch Conway? J. Am. Philos. Assoc. 4 (3), 280–297. 10.1017/apa.2018.24

[B14] Gunkel D. Wales J. J. (2021). Debate: What Is Personhood in the Age of AI? AI Soc. 36, 473–486. 10.1007/s00146-020-01129-1

[B15] Hermitte M-A. (1999). Le Droit Est Un Autre Monde. Enquête [En ligne]. Available at: http://journals.openedition.org/enquete/1553 (Accessed July 15, 2013).

[B16] Hondius F. W. (1980). Data Law in Europe. Stanford J. Intern. Law 16, 87–112.

[B17] Jowitt J. (2021). Assessing Contemporary Legislative Proposals for Their Compatibility with a Natural Law Case for AI Legal Personhood. AI Soc. 36, 499–508. 10.1007/s00146-020-00979-z

[B18] Kiršienė J. Gruodytė E. Amilevičius D. (2021). From Computerised Thing to Digital Being: mission (Im)possible? AI Soc. 36, 547–560. 10.1007/s00146-020-01051-6

[B20] Kruger J. (2021). “Nature, Culture, AI and the Common Good – Considering AI’s Place in Bruno Latour’s Politics of Nature,” in Artificial Intelligence Research, First Southern African Conference for AI Research, SACAIR 2020, Muldersdrift, South Africa, 22–26, Proceedings. Editor Gerber A. (Springer), 21.

[B45] Kurki V. A. J. (2019). A Theory Of Legal Personhood. Oxford: Oxford University Press

[B21] Latour B. (2004). Politics of Nature – How to Bring the Sciences into Democracy. Cambridge: Harvard University Press.

[B22] Laukyte M. (2021). The Intelligent Machine: A New Metaphor through Which to Understand Both Corporations and AI . AI & Soc. 36, 445–456. 10.1007/s00146-020-01018-7

[B23] McPherson T. Plunkett D. (2020). “Conceptual Ethics and the Methodology of Normative Inquiry,” in Conceptual Engineering and Conceptual Ethics. Editors Burgess A. Cappelen H. Plunkett D. (Oxford University Press). 10.1093/oso/9780198801856.003.0014

[B24] Menary R. (2007). Cognitive Integration. UK: Palgrave Macmillan.

[B25] Michalczak R. (2017). “Animals' Race against the Machines,” in Legal Personhood: Animals, Artificial Intelligence and the Unborn (Springer, Law and Philosophy Library), 91–101. 10.1007/978-3-319-53462-6_6

[B11] Norton D. F. Norton M. J. (Editors) (2007). David Hume, A Treatise of Human Nature: A Critical Edition (Oxford, Clarendon Press), 27.

[B44] Novelli C. Bongiovanni G. Sartor G. (2021). A Conceptual Framework For Legal Personality And Its Application To AI. Jurisprudence. 10.1080/20403313.2021.2010936

[B26] Papakonstantinou V. de Hert P. (2020). Refusing to Award Legal Personality to AI: Why the European Parliament Got it Wrong, European Law Blog. Available at: https://europeanlawblog.eu/2020/11/25/refusing-to-award-legal-personality-to-ai-why-the-european-parliament-got-it-wrong/.

[B27] Pietrzykowski T. (2018). Personhood beyond Humanism - Animals, Chimeras, Autonomous Agents and the Law. Cham: Springer.

[B28] Pietrzykowski T. (2017). “The Idea of Non-personal Subjects of Law,” in Legal Personhood: Animals, Artificial Intelligence and the Unborn (Springer, Law and Philosophy Library). 10.1007/978-3-319-53462-6_4

[B29] Pottage A. Mundy M. (2004). Law, Anthropology, and the Constitution of the Social, Making Persons and Things. Cambridge University Press.

[B30] Pound R. (1922). An Introduction to the Philosophy of Law. Yale University Press, 16.

[B31] Regad C. Riot C. (2020). La personnalité juridique de l'animal (II) : Les animaux liés à un fonds (de rente, de divertissement, d'expérimentation). Toulon: LexisNexis.

[B32] Regad C. Riot C. (2018). Sylvie Schmitt, La personnalité juridique de l'animal (I) : L'animal de compagnie. Toulon: LexisNexis.

[B33] Rizoiu R. (2020). “În Spatele Oglinzii: Voința Ca Putere,” in Dreptul romanesc la 100 de ani de la Marea Unire. Editors Pop, P. Rizoiu R. (București: Editura Hamangiu), 579.

[B35] Schirmer J-E. (2020). “Artificial Intelligence and Legal Personality: Introducing “Teilrechtsfähigkeit”: A Partial Legal Status Made in Germany,” in Regulating Artificial Intelligence. Editors Wischmeyer, T. Rademacher T. (Springer), 124.

[B36] Solum L. B. (1992). Legal Personhood for Artificial Intelligences. North Carolina L. Rev. 70, 1231–1288.

[B37] Sousa Antunes H. (2020). Civil Liability Applicable to Artificial Intelligence: a Preliminary Critique of the European Parliament Resolution of 2020. Available at: https://ssrn.com/abstract=3743242.

[B38] Teubner G. (2018). Digital Personhood? the Status of Autonomous Software Agents in Private Law, Translated by Jacob Watson. Ancilla Juris, 35–78.

[B39] Tuori K. (2002). Critical Legal Positivism. London: Ashgate, 186–188.

[B40] Kurki, V. A. J. Pietrzykowski T. (Editors) (2017). Legal Personhood: Animals, Artificial Intelligence And the Unborn (Springer), viii.

[B41] Veale M. Zuiderveen B. F. (2021). Demystifying the Draft EU Artificial Intelligence Act — Analysing the Good, the Bad, and the Unclear Elements of the Proposed Approach. Comp. L. Rev. Int. 22 (4), 97–112. 10.9785/cri-2021-220402

[B42] Wein L. E. (1992). The Responsibility of Intelligent Artifacts: Toward an Automation Jurisprudence. Harv. J. L. Tech. 6, 103–154.

[B43] Wojtczak S. (2021). Endowing Artificial Intelligence with Legal Subjectivity. AI & Soc. 10.1007/s00146-021-01147-7

